# Identification of Rabbit Annulus Fibrosus-Derived Stem Cells

**DOI:** 10.1371/journal.pone.0108239

**Published:** 2014-09-26

**Authors:** Chen Liu, Qianping Guo, Jun Li, Shenghao Wang, Yibin Wang, Bin Li, Huilin Yang

**Affiliations:** 1 Department of Orthopaedics, The First Affiliated Hospital of Soochow University, Suzhou, Jiangsu, China; 2 Orthopedic Institute, Soochow University, Suzhou, Jiangsu, China; National Centre for Scientific Research, ‘Demokritos’, Greece

## Abstract

Annulus fibrosus (AF) injuries can lead to substantial deterioration of intervertebral disc (IVD) which characterizes degenerative disc disease (DDD). However, treatments for AF repair/regeneration remain challenging due to the intrinsic heterogeneity of AF tissue at cellular, biochemical, and biomechanical levels. In this study, we isolated and characterized a sub-population of cells from rabbit AF tissue which formed colonies *in vitro* and could self-renew. These cells showed gene expression of typical surface antigen molecules characterizing mesenchymal stem cells (MSCs), including CD29, CD44, and CD166. Meanwhile, they did not express negative markers of MSCs such as CD4, CD8, and CD14. They also expressed Oct-4, nucleostemin, and SSEA-4 proteins. Upon induced differentiation they showed typical osteogenesis, chondrogenesis, and adipogenesis potential. Together, these AF-derived colony-forming cells possessed clonogenicity, self-renewal, and multi-potential differentiation capability, the three criteria characterizing MSCs. Such AF-derived stem cells may potentially be an ideal candidate for DDD treatments using cell therapies or tissue engineering approaches.

## Introduction

As the major cause of low back pain which affects about 80% of the population, degenerative disc disease (DDD) has evolved into a serious medical problem and significantly contributes to healthcare costs [1]. Tissue engineering has emerged as a promising approach toward DDD therapy [2]. As a component which plays a critical role in the biomechanical properties of intervertebral disc (IVD), the annulus fibrosus (AF) is essential for confining nucleus pulposus (NP) and maintaining physiological intradiscal pressure [2]. However, despite recent advancements [3]–[6], major challenge remains toward AF tissue engineering, mainly due to the tremendous complexity of AF tissue at cellular, biochemical, microstructural, and biomechanical levels [7], [8].

Cells play a central role in determining the quality of engineered tissues. Currently, tissue engineering of AF mainly involve the use of AF cells [4], [9], [10], chondrocytes [5], or bone marrow stem cells (BMSCs) [3], [6] of various origins. However, due to the ageing of differentiated cells, low cellularity, and the intrinsic phenotype heterogeneity of AF cells, application of AF cells or chondrocytes for AF repair/regeneration is limited [11], [12]. Use of BMSCs, which have been overwhelmingly used and shown effectiveness in AF tissue engineering, also confronts with a problem of limited cell availability (only 0.001–0.01% BMSCs in bone marrow aspirates or marrow tissue) [13]. Therefore, seeking new cell sources for AF tissue engineering appears to be necessary.

To date, mesenchymal stem cells (MSCs) have been isolated from a variety of adult tissues and they differ in many ways [14]. As a rule of thumb, MSCs from adult tissues tend to be tissue specific, meaning that MSCs originated from a certain tissue preferentially differentiate into the type of cells residing in this tissue [14]–[17]. Recently, it has been suggested that stem cell niches are present at the border of the AF and that the stem cells or progenitor cells migrate into the AF [15], [16], [18]. There have been several lines of evidence implying that stem/progenitor cells exist in AF, such as formation of cartilage, bone, and nerve tissues in AF during IVD degeneration, likely as a result of the differentiation of progenitor cells in AF or NP [8], [15], [19]–[21]. Such stem/progenitor cells, if successfully isolated, may be a valuable source for AF cell therapy and tissue engineering due to their AF tissue specificity.

To this end, this study aimed to isolate and characterize stem cells from AF tissue. Such stem cells should possess clonogenicity, self-renewal capability, and multipotency, the common characteristics of MSCs [22]. Since rabbit is a commonly used model for IVD research taking advantage of its moderate size, ease of surgery, and post-surgery analyses [15], [16], [23], we used rabbit IVDs to isolate a population of AF-derived colony-forming and characterize the properties of these cells. As expected, we found that these cells could self-renew and be readily induced to differentiate into osteocytes, chondrocytes, and adipocytes. Such findings revealed the existence of AF-derived stem cells, which may potentially be a valuable source for repair or regeneration of AF tissue.

## Materials and Methods

### Isolation of AF-derived Cells

AF samples were isolated from IVDs of female New Zealand white rabbits (6–8 weeks old) (**Fig. S1**) and minced and digested using 150 U/ml Collagenase I (Sigma, Cat.# C0130) in DMEM-LG medium for 4–6 hr. The suspension was then centrifuged at 1000 rpm for 10 min. The cell pellet was re-suspended in DMEM-LG supplemented with 20% FBS, 100 U/ml penicillin,100 µg/ml streptomycin and plated in 100 mm tissue culture dishes. Cells were maintained in a humidified incubator at 37°C with 5% CO_2_. The medium was changed every 2 days until the cells reached sub-confluence, then cells were harvested using 0.25% trypsin-EDTA. Isolation of spleen cells as a control for RT-PCR analysis was described in the file **Methods S1**. The animal surgery protocols were approved by the Institutional Animal Care and Use Committee (IACUC) of Soochow University.

### Colony Forming Assay

To test the colony forming capability of AF-derived cells, a colony forming unit-fibroblast (CFU-F) assay was performed. Highly diluted single cell suspensions with cell densities of 200–5,000 cells/ml were plated and cultured for 10 days. Then the cells were washed and fixed with 4% paraformaldehyde for 15 minutes. After further rinsing twice with PBS, cells were stained with 0.07% crystal violet (in ethanol) at 4°C and the colonies were counted.

### Polymerase Chain Reaction for Gene Analysis

Total RNA was extracted from cells using Trizol reagent (Invitrogen, Cat.# 15596-026) and then reversely transcribed to cDNA using a reverse transcription kit (Fermentas, Cat.# K1622) after DNase I treatment (Fermentas, Cat.# EN0521). The genes included MSC positive marker genes (CD29, CD44, CD166), MSC negative marker genes (CD4, CD8, CD14), adipocyte specific genes (peroxisome proliferators-activated receptor γ, Lipoprotein lipase), osteocyte specific genes (collagen type I, Runx 2), chondrocyte specific genes (collagen type II, Sox-9) and housekeeping gene GAPDH. All the primer sequences are listed in **Table 1**. The sequences were designed according to published papers or designed by ourselves. All the primers were synthesized by Invitrogen.

**Table 1:** Sequences of primers for RT-PCR.

Gene	Size (bp)	Primer sequence	Type	Tm (°C)	Gene Bank #
CD29	242	5′-GTCACCAACCGTAGCAA-3′	Forward	58	AY195896.1
		5′-CTCCTCATCTCATTCATCAG-3′	Reverse		
CD44	191	5′-CGATTTGAATATAACCTGCCGC-3′	Forward	63	FJ360436.1
		5′-CGTGCCCTTCTATGAACCCA-3′	Reverse		
CD166	200	5′-GGACAGCCCGAAGGAATACGAA-3′	Forward	63	Y13243.1
		5′-GACACAGGCAGGGAATCACCAA-3′	Reverse		
CD4	273	5′-GATGGAGGTGGAACTGC-3′	Forward	63	NM_001082313.2
		5′-GGAAAGCCCAACACTATG-3′	Reverse		
CD8	126	5′-GGGTGGAAAAGGAGAAGC-3′	Forward	63	L22293.1
		5′-AGGTGAGTGCGGGAGAC-3′	Reverse		
CD14	364	5′-CAGGTGCCTAAGGGACT-3′	Forward	63	NM_001082195.2
		5′-AATAAAGTGGGAAGCGG-3′	Reverse		
GAPDH	107	5′-ACTTTGTGAAGCTCATTTCCTGGTA-3′	Forward	58	L23961
		5′-GTGGTTTGAGGGCTCTTACTCCTT-3′	Reverse		
PPARγ	130	5′-CATTTTCTCAAGCAACAGTC-3′	Forward	54	NM_001082148.1
		5′-CAAAGGAGTGGGAGTGGT-3′	Reverse		
LPL	487	5′-GGCGAGACGCACGAACA-3′	Forward	54	FJ429312.1
		5′-CACCCGCAGTACAAACCCA-3′	Reverse		
Col I	81	5′-CTGACTGGAAGAGCGGAGAGTAC-3′	Forward	58	AY633663
		5′-CCATGTCGCAGAAGACCTTGA-3′	Reverse		
Runx-2	154	5′- CAGGCAGTTCCCAAGCATTTCA-3′	Forward	67	AY598934
		5′- TGGTGGCAGGTAGGTATGGTAGT-3′	Reverse		
Col II	84	5′-TGGGTGTTCTATTTATTTATTGTCTTCCT-3′	Forward	62	S83370
		5′-GCGTTGGACTCACACCAGTTAGT-3′	Reverse		
Sox-9	261	5′-TACGACTGGACGCTGGTGC-3′	Forward	67	AY598935
		5′-CGGGTGGTCTTTCTTGTGCT-3′	Reverse		

### Immunofluorescence

Cells were fixed in cold 4% poly-formaldehyde for 15 min, followed with treatment using methanol at −20°C for 5 min. They were then blocked with 4% BSA for 30 min before being incubated with mouse anti-human Oct-4 antibody (1∶500, Millipore, Cat.# MAB4401), goat anti-human nucleostemin antibody (1∶250, Neuromics, Cat.# GT15050), and mouse anti-human SSEA-4 antibody (1∶200, Invitrogen, Cat.# 41-4000), for staining of Oct-4, nucleostemin, and SSEA-4, respectively. Following that, Cy3-conjugated secondary antibodies were applied. Nucleus staining was accomplished by DAPI staining. Cells were viewed under a fluorescence inverted microscope (EVOS f1, AMG, USA). The detailed protocols are provided in file **Methods S1**.

### Induced Differentiation

Passage 2–4 AF-derived cells were seeded at a density of 4×10^4^ cells/well in a 24 well plate in basic culture medium (DMEM-LG supplemented with 10% FBS, 100 U/ml penicillin,100 µg/ml streptomycin). They were subjected to induced differentiation by culturing them in osteogenic, chondrogenic, and adipogenic media, respectively. The outcomes were evaluated using Alizarin Red S, and Safranin O, and Oil Red O staining, respectively. The detailed protocols are shown in file **Methods S1**.

### Statistical Analysis

All quantitative data are presented as mean ± standard deviation (SD) with no less than three replicates for each experimental condition. Statistical analyses were performed using one-way analysis of variance (ANOVA). Difference with *p*<0.05 was considered statistically significant.

## Results

### Colony Formation and Proliferation of Rabbit AF-Derived Cells

**Figure 1:**
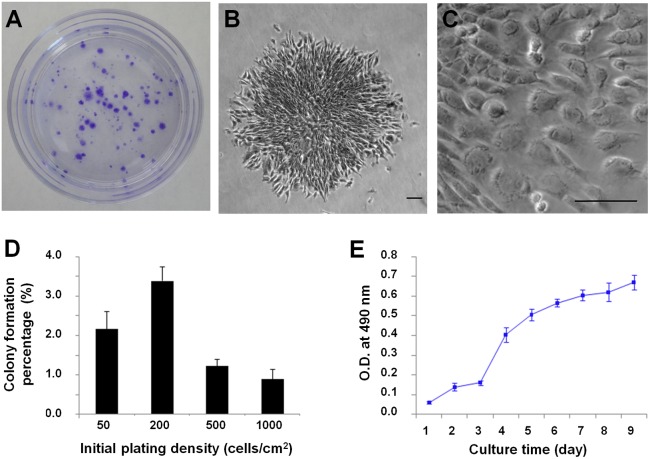
Colony formation and proliferation of rabbit AF-derived cells. (**A**) Total colonies stained with crystal violet at 10 days. (**B**) A representative cell colony. (**C**) The morphology of cells in a colony. (**D**) Colony forming unit assay for AF-derived cells. (**E**) Growth curve of AF-derived cells at passage 3. Scale bars, 100 µm.

When the rabbit AF-derived cells were cultured in the growth medium supplemented with 20% FBS, they remained quiescent for 3 to 4 days and then started to form colonies (**Fig. 1**). These colonies differed in sizes with diameter ranging from 1 to 3 mm. The morphology of cells in the colonies also varied, with some of them being cobblestone-like and others being spindle-like (**Fig. S2** and **Fig. S3**). The colony formation capacity was largely dependent on the initial cell seeding density. An initial seeding density of 200 cells/cm^2^ was found to result in the highest efficiency of colony formation, in which about 3.4% of plated cells formed colonies (**Fig. 1D**). After 10 to 12 days of culture, cells reached sub-confluence and were harvested and sub-cultured. Cell proliferation capacity was tested using cells at passage three through MTT assay. It was clear that cells began to grow after 1 day and entered log phase at the 3^rd^ day (**Fig. 1E**). The typical population doubling time was 17.8 hrs, indicating the strong self-renewing capacity of the rabbit AF-derived colony-forming cells.

### Expression of Stem Cell Markers in AF-Derived Colony-Forming Cells

**Figure 2:**
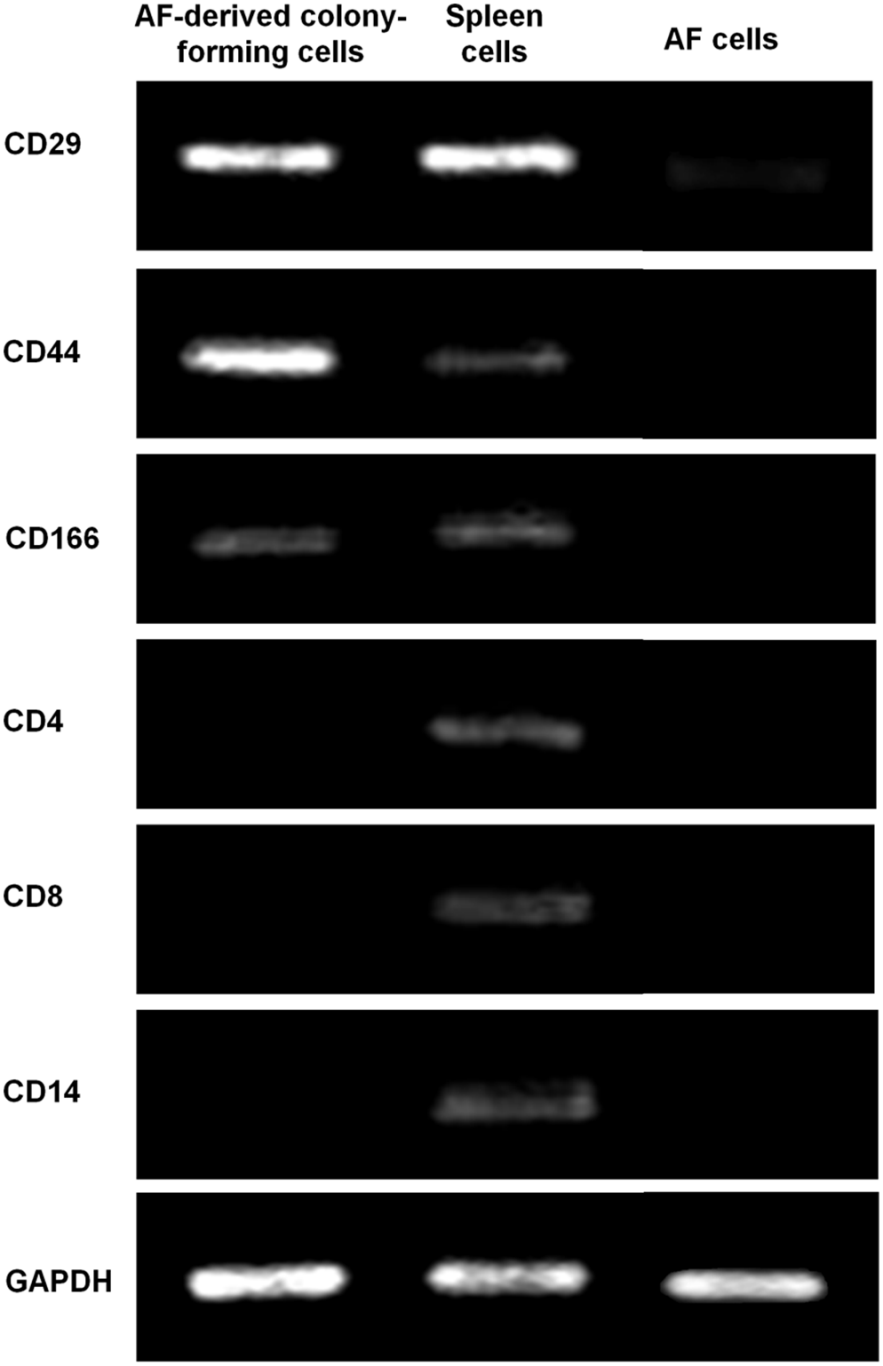
Expression of stem cell markers in AF-derived colony forming cells. AF-derived colony forming cells, but not AF cells, were positive for CD29, CD44, CD166, but negative for CD4, CD8 and CD14. As a positive control, spleen cells expressed all these genes.

The gene expression of typical MSC-associated surface antigens in cells was tested using RT-PCR. Clearly, the AF-derived colony-forming cells had strong expression of markers that are usually positive in MSCs, including CD29, CD44 and CD166. Meanwhile, they had little expression of markers which seldom exist in MSCs, including CD4, CD8 and CD14 (**Fig. 2**). As a control, cells from rabbit spleen were found to express all the above genes, which validated the efficacy of the customer-designed primers.

**Figure 3:**
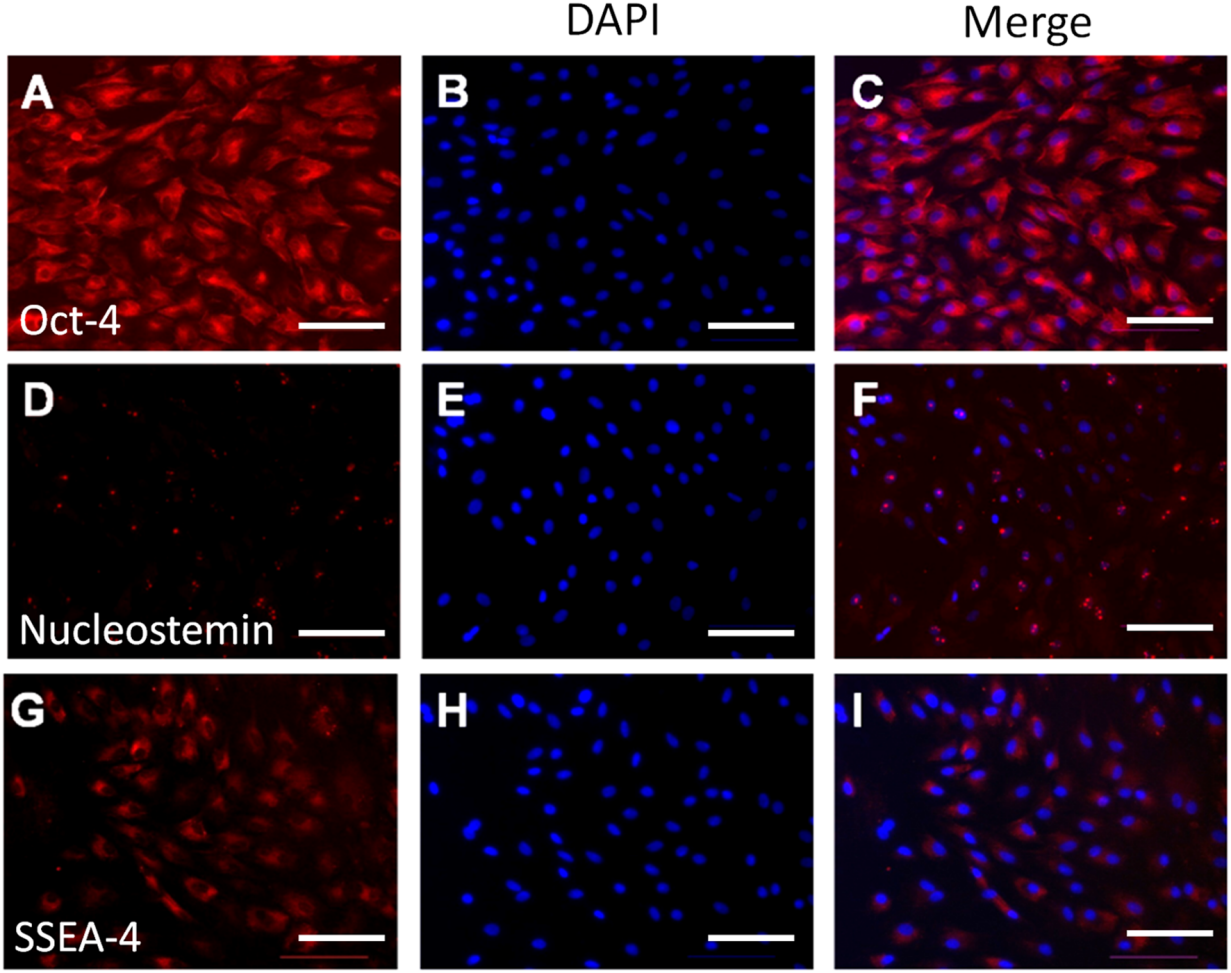
Immunofluorescence assays for stem cell marker protein expression in AF-derived colony forming cells. The AF-derived cells were positive for Oct-4 (**A–C**), nucleostemin (**D–F**) and SSEA-4 (**G–I**). Scale bars, 200 µm.

Further, the expression of MSC markers including Oct-4, nucleostemin (NS) and SSEA-4 was examined at protein level using immunofluorescence. As shown in **Fig. 3**, these markers were extensively expressed in the AF-derived cells. Among them, Oct-4 was located both within the nucleus and throughout the cytoplasm; nucleostemin existed exclusively within the nucleus; whereas SSEA-4 was presented within the cytoplasm.

### Multi-Potential Differentiation AF-Derived Colony-Forming Cells

**Figure 4:**
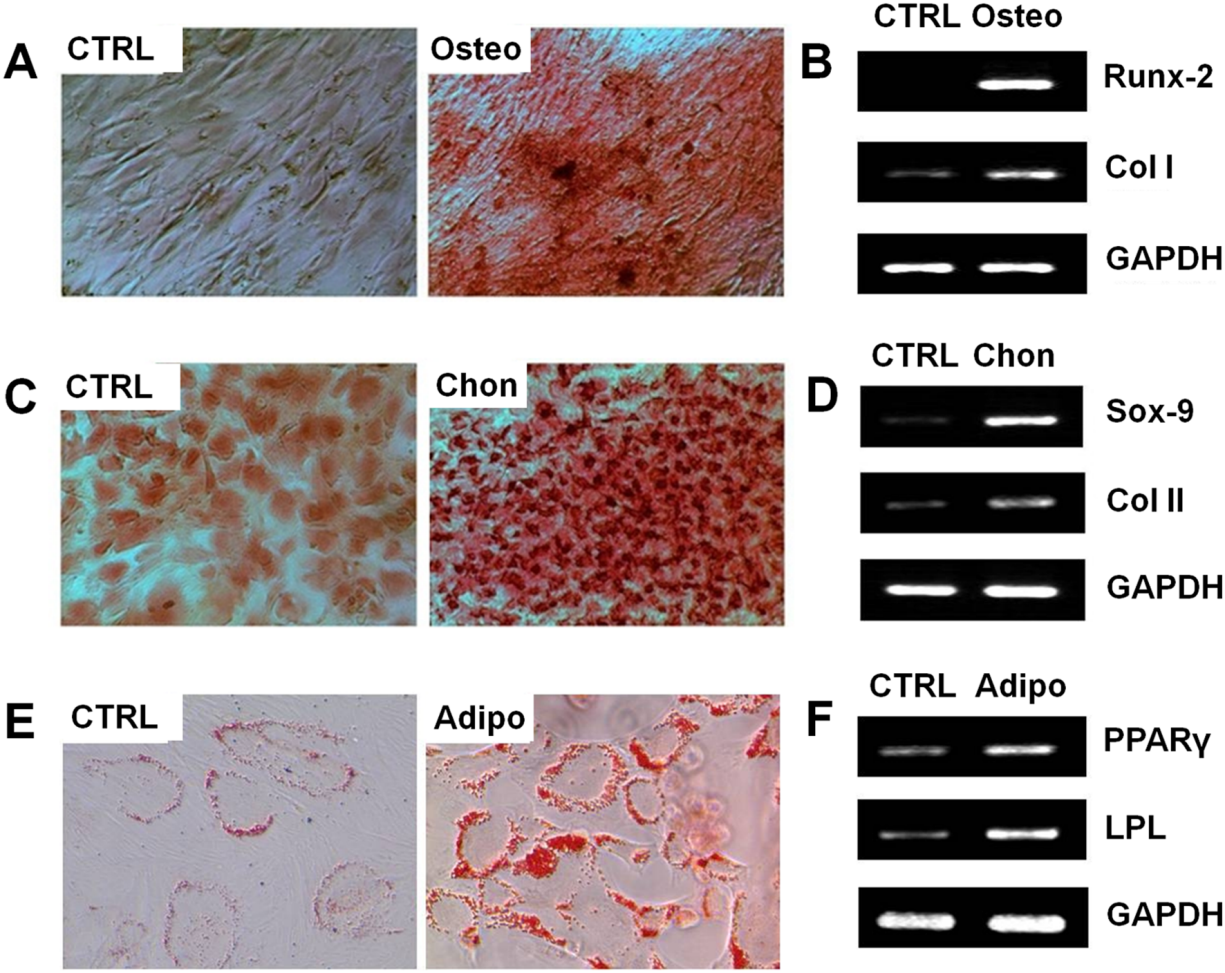
Induced differentiation of AF-derived colony forming cells. (**A**–**B**) Osteogenic differentiation at 3 weeks. Mineralization was stained with Alizarin red S (**A**). Expression of osteocyte-specific genes, including *Runx-2* and *Col I*, were up-regulated in induced cells as analyzed by RT-PCR (**B**). (**C**–**D**) Chondrogenic differentiation at 3 weeks. Cells were stained with Safranin O (**C**). Expression of chondrocyte-specific genes *Sox-9* and *Col II* levels was higher in induced cells (**D**). (**E**–**F**) Adipogenic differentiation at 2 weeks. Secretion of oil droplets (**E**) and expression of adipocyte-specific genes *PPAR-γ* and *LPL* (**F**) were higher in induced cells.

Next, the rabbit AF-derived colony-forming cells were subjected to induced differentiation processes including osteogenesis, chondrogenesis, and adipogenesis to examine the multi-differentiation potential of them. When the cells were cultured in osteogenic medium, their morphology started to change at the 5^th^ day. After 3 weeks, calcium deposits were highly visible in the induced cells which were fixed and stained with Alizarin Red S (**Fig. 4A**). In contrast, calcium deposits were rarely seen in control cells which were cultured in basic medium only. In addition, the mRNA expression of osteocytes-specific genes, including *Runx-2* and *Col I*, was examined using RT-PCR. Clearly, expression of both genes was higher in induced cells, indicating that the rabbit AF-derived cells had undergone successful osteogenesis (**Fig. 4B**).

When the AF-derived cells were cultured in chondrogenic differentiation medium, they round up and became chondrocyte-like cells. Strong production of sulfated proteoglycans in the induced cells after 3 weeks was visualized by Safranin O staining (**Fig. 4C**). The control cells cultured in basic medium also showed considerable Safranin O staining, albeit in a much lower level. At mRNA level, expression of chondrocytes-specific genes *Sox-9* and *Col II* was markedly up-regulated in induced cells compared to control cells (**Fig. 4D**).

During adipogenic differentiation, the rabbit AF-derived cells started to secrete small oil droplets, which surrounded the cells and constantly accumulated throughout the induced culture period, after 1 week of induction (**Fig. 4E**). Meanwhile, the expression of adipocyte-specific genes, including *PPAR-γ* and *LPL*, was also markedly enhanced in induced cells compared to control cells (**Fig. 4F**).

## Discussion

AF plays an important role in maintaining the physiological structure and function of IVD and its failure contributes to the evolution of DDD [24]. Cell-based therapies, from mere cell injection to tissue engineering, appear to be a promising approach to AF tissue repair or regeneration. Among the several types of cells that have been used for this purpose, MSCs remain to be the most used one because of their easy availability. However, there has been increasing consensus that the differentiation potential of adult MSCs largely varies and is closely dependent on their origin. For instance, synovium-derived MSCs showed better chondrogenic potential compared to MSCs derived from other types of tissues. Adipose-derived MSCs had higher adipogenic potential, while periosteum- and muscle-derived MSCs had better osteogenic potential [14]. Tendon MSCs also preferentially differentiated into tenocyte-like cells [25], [26]. Together, such studies indicate that adult MSCs originated from a certain tissue preferentially differentiate into the type of cells residing in this tissue [14], [27]. Such tissue-specific stem cells, therefore, are more appropriate cell source for cell therapies and tissue engineering of the specific tissues.

In light of this, we tried to isolate stem cells from rabbit AF, which may potentially be an ideal cell source for cell therapies and tissue engineering of AF, using single-cell colony forming technique. We found that in the best situation, about 3.4% of seeded primary cells formed colonies. Such colony formation efficiency is consistent to those found in other tissues such as tendon [25], [26]. The size of colonies and morphology of cells within colonies were heterogeneous, implying that different sub-populations of progenitor cells existed in AF. Such heterogeneity of colony-forming cells echoes the intrinsic cellular heterogeneity within AF. For example, cells at inner AF are chondrocytes-like, while cells at outer AF are fibroblasts-like, contributing to the region-dependent matrix composition of AF [28]. Cell proliferation tests indicated that these AF-derived colony-forming cells were capable of rapid growth, with a typical population doubling time of less than 18 hr. Such cell proliferation capability was kept until at least passage 6, making it possible for *in vitro* cell expansion to obtain sufficient number of AF cells for desired treatments. It should be noted that the regenerative capacity of stem cells markedly deteriorates with the aging of a living organism. Both the self-renewal and differentiation capabilities of adult stem/progenitor cells decrease with the age of animals [29]–[31]. It is expected that the number of stem/progenitor cells in more mature rabbits is less compared to those in younger species and their functional fitness also weakens. Therefore, young rabbits (6–8 weeks old) were used in this study to assure appropriate characterizations of the AF-derived colony-forming cells.

Further, we checked whether the AF-derived colony-forming cells expressed typical surface antigens of MSCs. Due to the very limited availability of anti-rabbit antibodies, we did not evaluate the protein expression of MSC surface markers using flow cytometry. Instead, we examined the expression of these markers at gene level using RT-PCR. We found that these cells expressed the positive markers of MSCs, including CD29, CD44 and CD166 [32], whereas they seldom expressed the negative markers such as CD4, CD8 and CD14 [33]. As a control, gene expression of cells isolated from rabbit spleen tissue was also examined. These cells were positive for CD4, CD8 and CD14 since spleen tissue contains lymphocytes and monocytes which express such markers [34]. Besides, these cells also expressed positive markers of MSCs [35].

In addition to surface antigens, we also examined the expression of several MSC marker proteins using immunofluorescence to confirm the stemness of AF-derived colony forming cells. Among these markers, Oct-4 is a transcription factor which is essential for maintaining undifferentiated status of pluripotent stem cells by inhibiting tissue-specific genes yet promoting stem cell-specific genes [36]. Nucleostemin is a nucleolar GTP-binding protein which is exclusively expressed in the nucleoli of stem cells, but not in committed and terminally differentiated cells, and is required in maintenance of self-renewal and pluripotency by controlling cell-cycle progression [37]. SSEA-4 is an early embryonic glycolipid antigen which is commonly used as human embryonic stem cell marker and also identifies adult MSCs [38]. We found that the AF-derived cells expressed all these stem cell markers, indicating their undifferentiated status (**Fig. 3**). It is interesting to note that Oct-4 was found to be presented throughout the whole cell instead of being located only in the nucleus as reported in previous studies [39]. Such a seemingly inconsistency may be the result of difference in cell type and origin, as Oct-4 has been found to be expressed in the whole cell of many somatic cells and cancer cells, including human kidney, gastric, mesenchymal, HeLa and MCF-7 cells [40]. In addition, even in the same type of stem cells, the distribution of Oct-4, in nucleus alone or throughout the cytoplasm, may significantly vary depending on the type of commercial antibodies [41].

In this study, we found that the rabbit AF-derived colony forming cells could undergo osteogenesis, chondrogenesis, and adipogenesis and therefore possessed multi-differentiation potential. Such results partially echo the findings from several previous studies based on human AF-derived cells [19], [20]. However, our study differs from these studies in that we have, for the first time, specifically isolated the cells that formed colonies, which are presumably stem/progenitor cells of reasonable purity. It is notable that in the chondrogenic test, the control cells, i.e., cells without being subjected to chondrogenic induction, also showed considerable Safranin O staining as well as expression of *Sox-9* and *Col II* genes (**Fig. 4C–D**). This is indeed an indication that such AF-derived colony forming cells have intrinsic tendency to commit to chondrocyte-like cells which constitute the major cell population residing in the fibrocartilage-like AF tissue. Thus, such findings strongly support the significant potential of AF-derived colony forming cells for AF therapies and tissue engineering. It should be noted that without *in vitro* expansion, the AF-derived colony forming cells are generally too few to be directly used for cell therapies. However, after serial passaging a considerable number of cells may be obtained. For example, we usually obtained 20–30 million cells from each rabbit AF segment after three passages, which is enough for a typical cell therapy (0.4–10 million per kg of body weight) [42]. Importantly, the cells showed no apparent difference in morphology, proliferation capacity, stem marker expression and differentiation potential as long as they were within passage six (data not shown). In humans, AF tissues may be obtained through surgeries in which the disc is partially or entirely removed, including discectomy, interbody fusion, or scoliosis treatments [24]. Following that, the AF-derived colony forming cells can be retrieved and expanded for cell therapy or tissue engineering applications, as implied in previous studies [19], [20].

In addition, the differentiation potential was independent of the morphology of colony. For example, both chondroblast-like and fibroblast-like colonies could undergo induced differentiation. This implies that while different cell sub-populations may exist in colony forming cells derived from different AF regions, they all have the potential to differentiate into AF cells or various regions. It again signifies the importance of using such colony forming cells for repairing/regenerating the AF tissue, which is intrinsically a heterogeneous tissue and has distinct region-specific characteristics. It should be noted, however, that compared to cells from fibroblast-like colonies, the cells from chondroblast-like colonies showed relatively stronger tendency to differentiate to chondrocyte-like cells, as indicated by the stronger Safranin O staining as well as *Sox-9* and *Col II* gene expression (data not shown). In addition to induced differentiation tests, we found another piece of evidence of the plasticity of AF-derived colony forming cells, i.e., substrate elasticity-dependent differentiation characteristics [43]. When they were cultured on substrates of various stiffness, their gene expression profiles varied in a stiffness-dependent manner. The cells became more chondrocyte-like on relatively compliant substrate with up-regulated *Col II* gene expression, while they became more fibroblast-like on stiffer substrate and markedly expressed more *Col I* gene (**Fig. S4**).

In summary, we have successfully isolated and identified a population of colony forming cells from rabbit AF tissue. These cells possess clonogenicity, self-renewing capability, and multi-differentiation potential, the common characteristics of MSCs. Therefore, we define these cells as AF-derived stem cells (AFSCs). Being capable of rapid *in vitro* expansion and multi-differentiation, the AFSCs may potentially be a valuable cell source for DDD treatments using cell therapy or tissue engineering approaches. It should be noted, however, that findings in this study do not rule out the feasibility of using other cell sources for AF tissue regeneration. In another study, we have found that after certain treatment such as TGF-β3, BMSCs can also be a good cell source for AF regeneration (unpublished data). Future research will explore the lineage commitment of AFSCs toward various types of cells within AF and compare the efficiency as cell source in AF tissue engineering between AFSCs and other commonly used MSCs, including bone marrow stem cells and adipose-derived stem cells.

## Supporting Information

Figure S1 **Harvest of rabbit AF tissues.** (A) A segment of spinal column containing IVDs from T10 through L5 vertebra. (B) IVD harvesting. (C) A whole rabbit IVD. The AF tissue was then separated from the IVD by removing the nucleus pulposus.
(TIF)

Figure S2 **Cell colonies formed from rabbit AF cells using single cell culture technique.** (A–B) Typical colonies formed from single rabbit AF cells. The cells were seeded at different initial plating densities and were stained at 10 days using crystal violet. (C–D) Gross view of the morphology of cells within cell colonies of various sizes. Scale bars, 100 µm.
(TIF)

Figure S3 **Representative close view of the morphology of AF cells within colonies. (**A–B) Cells from the inner and outer regions, respectively, of one colony. (C–D) Cells from the inner and outer regions, respectively, of another colony. The cells were stained with crystal violet.
(TIF)

Figure S4 **Expression of** ***Col I*** **(A) and** ***Col II*** **(B) genes in rabbit AF-derived colony forming cells cultured on polyacrylamide hydrogels of different Young’s moduli for 1 week.**
(TIF)

Methods S1 **Supporting methods.**
(DOCX)
