# Supplementary figures and images for: Identification of Rabbit Annulus Fibrosus-Derived Stem Cells
Source: PLoS One. 2014 Sep 26;9(9):e108239. doi: 10.1371/journal.pone.0108239 (PMC4178129; doi:10.1371/journal.pone.0108239)

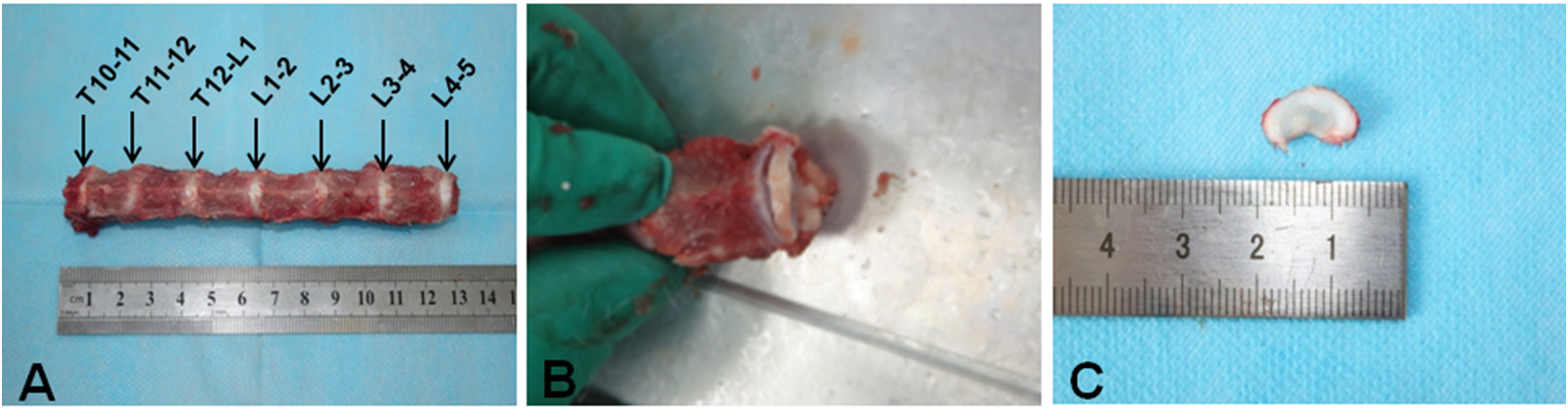

Supplement: Figure S1 — Harvest of rabbit AF tissues. (A) A segment of spinal column containing IVDs from T10 through L5 vertebra. (B) IVD harvesting. (C) A whole rabbit IVD. The AF tissue was then separated from the IVD by removing the nucleus pulposus. (TIF) [file pone.0108239.s001.tif]

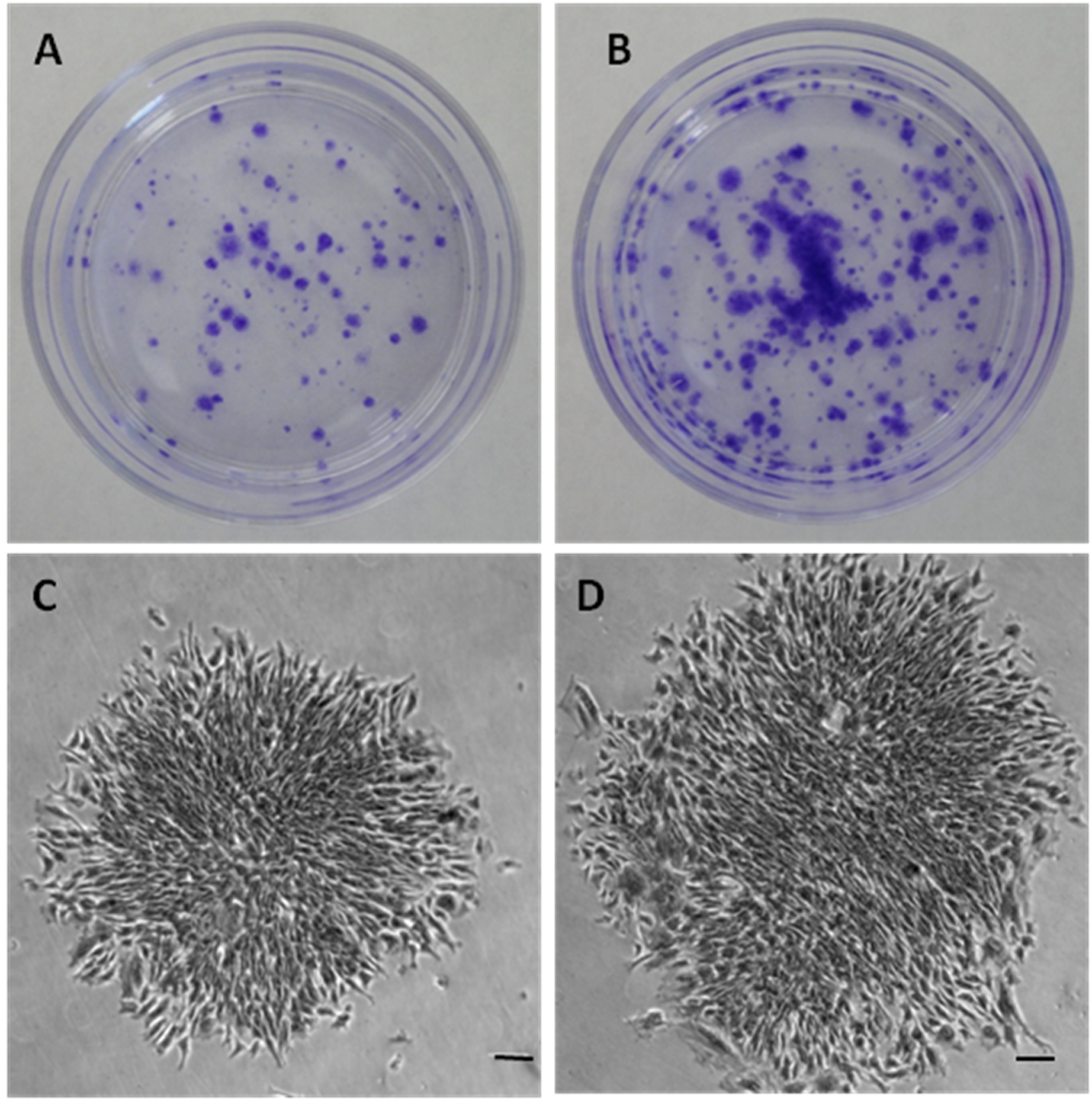

Supplement: Figure S2 — Cell colonies formed from rabbit AF cells using single cell culture technique. (A–B) Typical colonies formed from single rabbit AF cells. The cells were seeded at different initial plating densities and were stained at 10 days using crystal violet. (C–D) Gross view of the morphology of cells within cell colonies of various sizes. Scale bars, 100 µm. (TIF) [file pone.0108239.s002.tif]

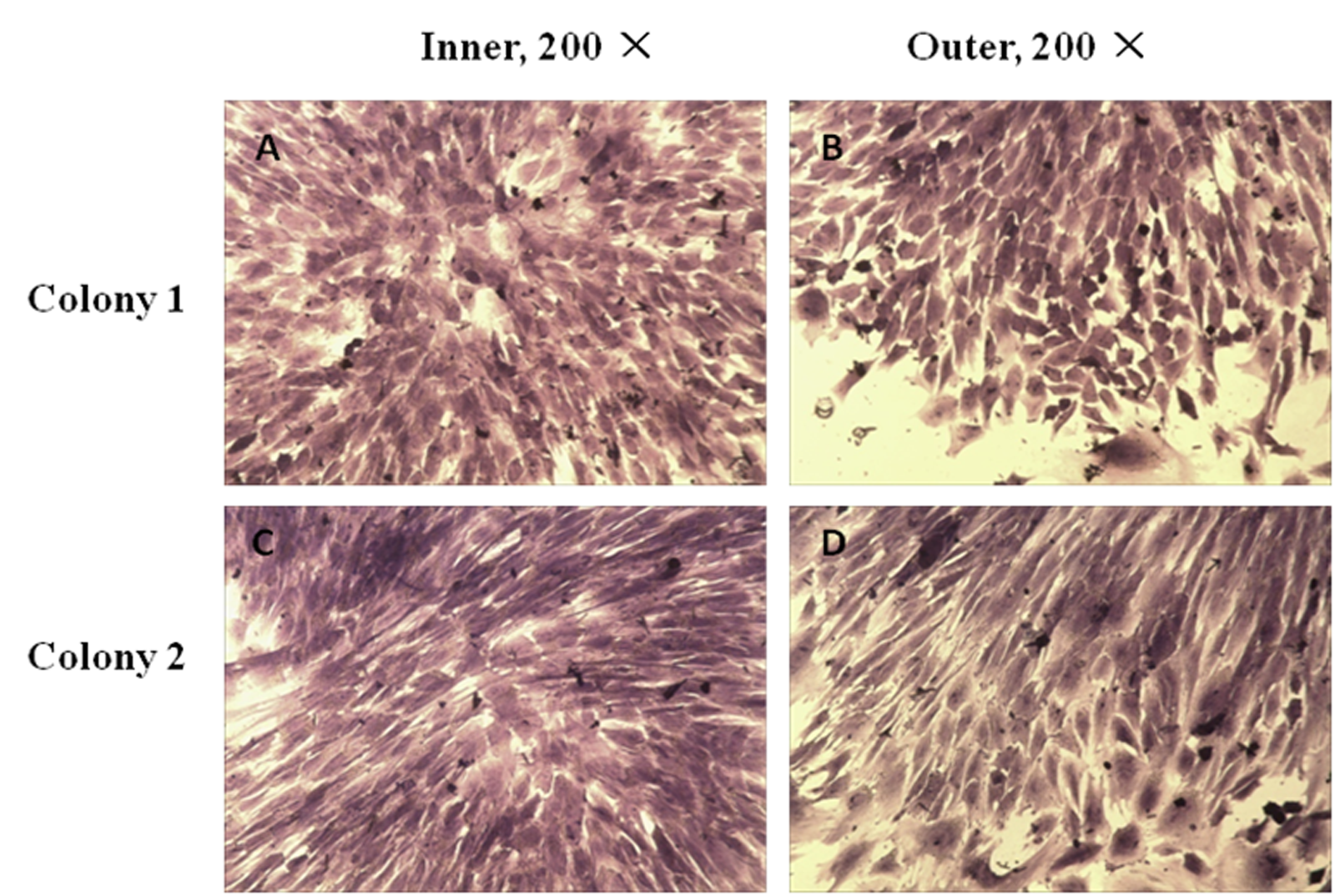

Supplement: Figure S3 — Representative close view of the morphology of AF cells within colonies. (A–B) Cells from the inner and outer regions, respectively, of one colony. (C–D) Cells from the inner and outer regions, respectively, of another colony. The cells were stained with crystal violet. (TIF) [file pone.0108239.s003.tif]

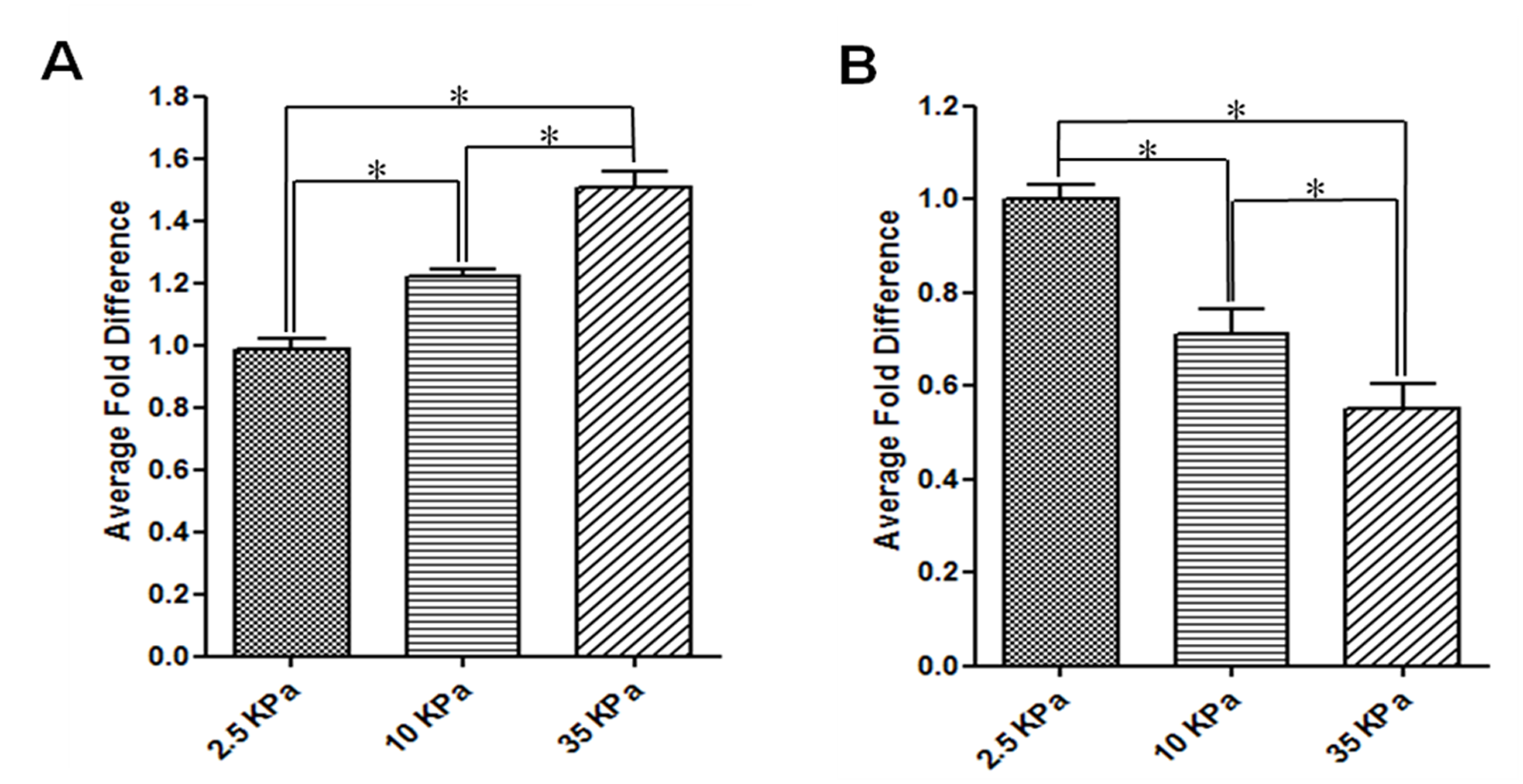

Supplement: Figure S4 — Expression of Col I (A) and Col II (B) genes in rabbit AF-derived colony forming cells cultured on polyacrylamide hydrogels of different Young’s moduli for 1 week. (TIF) [file pone.0108239.s004.tif]
