# Supplementary material for: Identification of Rabbit Annulus Fibrosus-Derived Stem Cells
Source: PLoS One. 2014 Sep 26;9(9):e108239. doi: 10.1371/journal.pone.0108239 (PMC4178129; doi:10.1371/journal.pone.0108239)
Supplement: Methods S1 — Supporting methods. (DOCX) [file pone.0108239.s005.docx]

**Methods S1**

**Isolation of Spleen Cells**

The spleen tissue was dissected from rabbit. To isolate total cells including lymphocytes, monocytes, splenocytes, spleen tissue was grinded by syringe in DMEM-LG and filtrated with 200 mesh stainless steel sieve to form single cell solution. To remove blood red cells, cell pellet by centrifuging at 1000 rpm for 10 min was treated with red blood cell lysis buffer. After centrifuge the remaining mixture of cells was used for total RNA isolation.[^1^](#_ENREF_1)

**Immunofluorescence**

Cells were fixed in cold 4% poly-formaldehyde for 15 min, followed with treatment using methanol at -20 °C for 5 min. For staining Oct-4, the cells were blocked with 4% BSA for 30 min before being incubated with mouse anti-human Oct-4 antibody (1:500, Millipore, Cat.# MAB4401) overnight at 4 °C. Then cells were incubated with Cy3-conjugated goat anti-mouse secondary antibody (1:1000, Invitrogen, Cat.# A10521) for 1 hr. Nucleus staining was accomplished by DAPI staining. Cells were viewed under a fluorescence inverted microscope (EVOS f1, AMG, USA). Similarly, goat anti-human nucleostemin antibody (1:250, Neuromics, Cat.# GT15050) along with Cy3-conjugated donkey anti-goat IgG secondary antibody (1:1000, Millipore, Cat.# AP180C) were used for nucleostemin. Mouse anti-human SSEA-4 antibody (1:200, Invitrogen, Cat.# 41-4000) along with Cy3-conjugated goat anti-mouse secondary antibody (1:1000, Invitrogen, Cat.# A10521) were used for SSEA-4 staining.

**Induced Differentiation**

Passage 2-4 AF-derived cells were seeded at a density of 4x10^4^ cells/well in a 24 well plate in basic culture medium (DMEM-LG supplemented with 10% FBS, 100 U/ml penicillin，100 μg/ml streptomycin). For adipogenesis, cells were induced in adipogenic medium consisting of basic culture medium supplemented with 1 uM dexamethasome (Sigma, Cat.# D4902), 10 μg/ml insulin (Sigma, Cat.# I6634), 100 uM indomethacin (Sigma, Cat.# I7378), and 0.5 mM isobutylmethylxanthine (IBMX) (Sigma, Cat.# I7018) after they reached full confluence for 2 days. For osteogenesis, cells were induced in osteogenic medium consisting of basic culture medium supplemented with 0.1 μM dexamethasone, 0.2 mM ascorbic-2-phospate (Sigma, Cat.# A8960), and 10 mM glycerol 2-phosphate (Sigma, Cat.# G8981) when cells reached 80% confluence. For chondrogenesis, cells were induced in chondrogenic medium consisting of basic culture medium supplemented with 40 μg/ml proline (Sigma, Cat.# P5607), 39 ng/ml dexamethasone, 10 ng/ml TGF-β 3 (Sigma, Cat.# T5425), 50 μg/ml ascorbate 2-phosphate, 100 μg/ml sodium pyruvate (Sigma, Cat.# P8574), and 50 mg/ml insuling-transferrin-selenious acid mix (ITS) (Sigma, Cat.# I1884) when cells reached 80% confluence. In control groups, all the cells were cultured in basic culture medium. Medium were changed every 3 days.

**Alizarin Red S Assay**

Osteogenesis was evaluated by Alizarin Red S staining. After the cells were cultured in osteogenic medium for 3 weeks, they were fixed in 4% paraformaldehyde for 40 min at room temperature, rinsed with PBS 3 times, and then stained with Alizarin Red S (Solarbio, Cat.# G8550 ) for 1 hr. The stained cells were examined using an inverted microscope.

**Safranin O Assay**

The chondrogenesis of AF-derived cells was evaluated using Safranin O staining. After the cells were induced by chondrogenic medium for 3 weeks, they were fixed with 4% paraformaldehyde for 1 hr. After washed with PBS for 3 times, the cells were stained with Safranin O (Sigma, Cat.# S2255) for 30 min. The stained cells were examined using an inverted microscope.

**Oil Red O Assay**

After culturing in adipogenic medium for 2 weeks, cells were fixed and stained with Oil Red O assay. The medium was aspirated and cells were washed with PBS for 3 times. Then the cells were fixed in 4% paraformaldehyde for 40 min at room temperature and subsequently stained with 0.36% Oil Red O (Sigma, Cat.# O0625) solution for 1 hr after being washed with PBS for 3 times. Finally the cells were carefully washed with ddH_2_O till the background was clean. The stained samples were observed using an inverted microscope.
